# Agreement Between Experts and an Untrained Crowd for Identifying Dermoscopic Features Using a Gamified App: Reader Feasibility Study

**DOI:** 10.2196/38412

**Published:** 2023-01-18

**Authors:** Jonathan Kentley, Jochen Weber, Konstantinos Liopyris, Ralph P Braun, Ashfaq A Marghoob, Elizabeth A Quigley, Kelly Nelson, Kira Prentice, Erik Duhaime, Allan C Halpern, Veronica Rotemberg

**Affiliations:** 1 Department of Dermatology Chelsea and Westminster Hospital London United Kingdom; 2 Dermatology Section Memorial Sloan Kettering Cancer Center New York, NY United States; 3 Department of Dermatology Andreas Syggros Hospital of Cutaneous and Venereal Diseases University of Athens Athens Greece; 4 Department of Dermatology University Hospital Zurich Zurich Switzerland; 5 Department of Dermatology The University of Texas MD Anderson Cancer Center Houston, TX United States; 6 Centaur Labs Boston, MA United States

**Keywords:** dermatology, dermatologist, diagnosis, diagnostic, labeling, classification, deep learning, dermoscopy, dermatoscopy, skin, pigmentation, microscopy, dermascopic, artificial intelligence, machine learning, crowdsourcing, crowdsourced, melanoma, cancer, lesion, medical image, imaging, development, feasibility

## Abstract

**Background:**

Dermoscopy is commonly used for the evaluation of pigmented lesions, but agreement between experts for identification of dermoscopic structures is known to be relatively poor. Expert labeling of medical data is a bottleneck in the development of machine learning (ML) tools, and crowdsourcing has been demonstrated as a cost- and time-efficient method for the annotation of medical images.

**Objective:**

The aim of this study is to demonstrate that crowdsourcing can be used to label basic dermoscopic structures from images of pigmented lesions with similar reliability to a group of experts.

**Methods:**

First, we obtained labels of 248 images of melanocytic lesions with 31 dermoscopic “subfeatures” labeled by 20 dermoscopy experts. These were then collapsed into 6 dermoscopic “superfeatures” based on structural similarity, due to low interrater reliability (IRR): dots, globules, lines, network structures, regression structures, and vessels. These images were then used as the gold standard for the crowd study. The commercial platform DiagnosUs was used to obtain annotations from a nonexpert crowd for the presence or absence of the 6 superfeatures in each of the 248 images. We replicated this methodology with a group of 7 dermatologists to allow direct comparison with the nonexpert crowd. The Cohen κ value was used to measure agreement across raters.

**Results:**

In total, we obtained 139,731 ratings of the 6 dermoscopic superfeatures from the crowd. There was relatively lower agreement for the identification of dots and globules (the median κ values were 0.526 and 0.395, respectively), whereas network structures and vessels showed the highest agreement (the median κ values were 0.581 and 0.798, respectively). This pattern was also seen among the expert raters, who had median κ values of 0.483 and 0.517 for dots and globules, respectively, and 0.758 and 0.790 for network structures and vessels. The median κ values between nonexperts and thresholded average–expert readers were 0.709 for dots, 0.719 for globules, 0.714 for lines, 0.838 for network structures, 0.818 for regression structures, and 0.728 for vessels.

**Conclusions:**

This study confirmed that IRR for different dermoscopic features varied among a group of experts; a similar pattern was observed in a nonexpert crowd. There was good or excellent agreement for each of the 6 superfeatures between the crowd and the experts, highlighting the similar reliability of the crowd for labeling dermoscopic images. This confirms the feasibility and dependability of using crowdsourcing as a scalable solution to annotate large sets of dermoscopic images, with several potential clinical and educational applications, including the development of novel, explainable ML tools.

## Introduction

The use of dermoscopy, a low-cost, noninvasive diagnostic technique based on a hand-held device with a light source and magnifying lens, is routine practice for the evaluation of pigmented skin lesions and has been shown to increase sensitivity for early melanoma detection [1,2]. Dermoscopy allows examination of morphological features below the stratum corneum that would not be visible by visual inspection alone [3]. Diagnosis of melanoma using dermoscopy relies on assessment of lesion morphology and identification of dermoscopic features. A number of diagnostic criteria and algorithms have been developed for this purpose, including pattern analysis [4], the ABCD (asymmetry, border, color, diameter) rule [5], the Menzies method [6], the 7-point checklist [7], and the CASH (color, architecture, symmetry, homogeneity) score [8].

As use of dermoscopy has expanded, so too has dermoscopic vocabulary, resulting in a vast number of published feature definitions and 2 competing terminologies: metaphoric and descriptive. In recent years, efforts have been made to harmonize nomenclature, and the 2016 International Dermoscopy Society terminology consensus proposed 31 specific “subfeatures” of melanocytic lesions, falling into 9 “superfeatures” based on structural similarities (Textbox 1) [9].

However, interrater reliability (IRR) for identifying melanoma-specific dermoscopic structures has been shown to be poor [10]. Our research group recently performed the EASY (Expert Agreement on the Presence and Spatial Location of Melanocytic Features in Dermoscopy) study, which found that agreement was highly variable when 20 dermoscopy experts were asked to identify the 31 dermoscopic subfeatures in an image set specifically curated for this purpose. IRR across 248 images was poor to moderate for all but 7 features. We demonstrated that when individual subfeatures were collapsed into 9 superfeatures, increased agreement was observed, ranging from a pairwise Fleiss κ of 0.14 for the detection of dots to 1.0 for the detection of a pigment network structure.

Machine learning (ML) methods have recently been investigated in the field of dermatology, and the majority of developed algorithms are diagnostic binary classifiers [11,12]. A number of studies have evaluated the performance of algorithms developed to detect specific dermoscopic features, including pigment network structures, vessels, and blue-white veil; however, many algorithms were trained and tested on relatively small data sets and have achieved only moderate accuracy [13-21].

Due to the vast dimensionality of medical images, classifier algorithms are typically of an uninterpretable “black box” nature, a term that describes the phenomenon whereby functions that connect input pixel data to output labels cannot be understood by the human brain. There has been a push by medical regulators and the artificial intelligence community to develop explainable algorithms; however, it has been acknowledged that this may come at the cost of decreased accuracy [22]. Incorporating detection of dermoscopic features into melanoma classifier algorithms may allow for better explainability and therefore greater acceptance into clinical practice by clinicians and regulatory bodies [23,24].

The International Skin Imaging Collaboration (ISIC) archive provides an open-access data set comprising almost 70,000 publicly available dermoscopic images at the time of writing, including 5598 melanomas and 27,878 nevi. As well as hosting the regular ISIC Grand Challenge to promote the development of ML for melanoma detection, the archive has been extensively utilized to train independent ML algorithms and acts as a comprehensive educational resource for dermatologists via the Dermoscopedia platform [25,26]. Most public images in the archive have labels serving as a diagnostic ground truth for supervised learning. However, accurate feature annotations are thus far lacking. As part of the 2018 ISIC Challenge, 2595 images were annotated for 5 dermoscopic patterns (pigment network structures, negative network structures, streaks, milia-like cysts, and dots/globules) [27]. However, the ground truth labels were provided by only 1 clinician and the performance of the 23 submitted algorithms was acknowledged to be exceptionally low, likely as a result of this [27].

As medical data sets continue to rapidly expand and computing power increases, it is widely recognized that one of the major limiting factors for the development of robust and generalizable ML in dermatology is the need for large, comprehensively labeled data sets [28,29]. Obtaining annotations of medical images by medical experts is both time-consuming and expensive, creating a bottleneck in the development pipeline and making it challenging to obtain annotations at scale [30].

Crowdsourcing provides a potential solution to these problems. Crowdsourcing involves the recruitment of groups of individuals of varying levels of knowledge, heterogeneity, and number who voluntarily complete an online task, often with financial incentives [31,32]. Monetary compensation is typically less than US $0.10 per annotation, and tasks can be distributed to a large number of workers in parallel, aggregating the crowd’s knowledge to complete the task in a cost- and time-effective manner [33,34]. One study reported that it took 6 months to obtain expert labels comprising 340 sentences from radiology reports written by 2 radiologists, whereas the authors obtained crowdsourced annotations of 717 sentences in under 2 days at a cost of less than $600. A classification algorithm trained using these crowdsourced annotations outperformed an algorithm trained using the expert-labeled data as a result of the increased volume of available training examples [32].

Given the heterogeneity of biomedical data, the utility of crowdsourcing may decrease with the complexity of the task. For example, the 14 million images contained in the ImageNet archive were easily annotated by the untrained public, whereas the ability to classify and segment radiological images may require many years of specialist training [28,30,35]. Nevertheless, crowdsourcing has proven effective in a wide range of applications for biomedical imaging, most commonly histopathology or retinal imaging [34].

Feng et al [36] reported that a crowd of South Korean students were able to reach similar diagnostic accuracy as experts for diagnosing malaria-infected red blood cells after only 3 hours of training, allowing the authors to build a gold standard library of malaria-infection labels for erythrocytes. The authors used a game-based tool that made the task easy to complete by including points and a leaderboard on the platform. This method of so-called gamification is frequently used by crowdsourcing platforms and has been shown to increase the engagement of the crowd and improve the quality of the crowdsourced work [37]. Bittel et al [38] used a hybrid crowd-ML approach to create the largest publicly available data set of annotated endoscopic images. Heim et al [28] found that a crowd was able to segment abdominal organs in computed tomography (CT) images with comparable quality to a radiologist, but at a rate up to 350 times faster.

There are few studies published to date evaluating crowdsourcing in the field of dermatology, and to the best of the authors’ knowledge, there are no published studies on the utility of crowdsourcing for the annotation of features present in dermoscopic images [39,40].

The aim of this study is to demonstrate that crowdsourcing can be employed to label dermoscopic subfeatures of melanocytic lesions with equivalent reliability to a small group of dermatologists. This will allow for efficient annotation of a large repository of dermoscopic images to aid the development of novel ML algorithms [32]. Incorporating detection of dermoscopic features into diagnostic algorithms will result in explainable outputs and may therefore improve the acceptability of these outputs to the medical community.

**List of superfeatures (in bold) and corresponding subfeatures seen in melanocytic lesions [9].**

**Dots**

Irregular, regular

**Globules**

Cobblestone pattern, irregular, regular, rim of brown globules

**Lines**

Branched streaks, pseudopods, radial streaming, starburst

**Network structures**

Atypical pigment network, broadened pigment network, delicate pigment network, negative pigment network, typical pigment network

**Regression structures**

Peppering/granularity, scarlike depigmentation

**Shiny white structures**

**Patterns**

Angulated lines, polygons, zigzags

**Structureless areas**

Irregular blotches, regular blotches, blue-whitish veil, milky red areas, structureless brown areas, and homogenous (not otherwise specified)

**Vessels**

Comma, corkscrew, dotted vessel, linear irregular vessel, polymorphous vessel, milky red globules

## Methods

### Ethics Approval

This study was conducted as part of the umbrella ISIC research protocol and was approved by the Memorial Sloan Kettering Cancer Center Institutional Review Board (16-974). All images were deidentified and do not contain any protected health information as per the terms of use agreement for the ISIC archive.

### Materials

This study was performed in 3 separate experiments, each using the same set of 248 lesion images used in the EASY study. Briefly summarized, clinical experts contributed 964 lesion images showing 1 of 31 preselected subfeatures, as described by Kittler et al [9]. Clinicians were asked to submit images of “excellent quality showing the exemplar feature in focus.” Three experts chose 248 of these images, roughly balancing benign and malignant lesions and ensuring image quality. Each of the 31 features was the exemplar in 8 of the lesion images submitted. However, each image could, and typically did, show multiple features.

### Subfeatures and Superfeatures

As described earlier, low to moderate IRR was observed for the majority of subfeatures. Hence, we used only the superfeature terms for our scalability investigation. While each of the subfeatures had 8 exemplar images, collapsing the labels into superfeatures created some imbalance. The full list of subfeatures is shown in Textbox 1. The 9 superfeatures (dots, globules, lines, network structures, patterns, regression structures, shiny white structures, structureless areas, and vessels; shown in Multimedia Appendix 1, Table S1) were presented to participants during the tutorial on the DiagnosUs smartphone app, adapted from Marghoob and Braun [41].

### Agreement Measure

To measure agreement across raters, we employed the Cohen κ [42], which has a value of 0 for completely random choices, increasing toward a maximum value of 1.0 with improved IRR. Measures of agreement are interpreted as poor (0-0.4), fair to good (≥0.4-0.75), and excellent (≥0.75-1.0) [43]. This measure was primarily chosen to accommodate the nature of the 3 separate studies (see below), allowing for partial data between pairs of raters using the binary choice of “feature present” or “feature absent.” Throughout this paper, we use the term “median κ” to refer to the median of κ values across the set of pairwise comparisons as a measure of central tendency, given the nonnormal distribution of κ values.

### Initial Expert Annotations (Study 1)

For the first study, we used a custom programmed annotation platform built for the ISIC archive. We asked a total of 20 dermoscopy experts to each annotate 62 images (2 per exemplar feature) in 4 substudies of nonoverlapping image sets. Experts for study 1 were clinicians with ≥10 years of dermoscopy experience who had made significant contributions to dermoscopy research or teaching dermoscopy of pigmented lesions. For each image, 5 experts were asked to provide benign/malignant status and then to self-select which of the 31 available subfeatures they perceived as present in the image. Full data and results of the EASY study will be published separately.

### Gold Standard for the Crowd Study

After collapsing the subfeatures into the 9 abovementioned superfeatures, we found that 3 had very poor agreement and too few exemplars to allow reliable evaluation by the crowd: patterns, shiny white structures, and structureless areas. For the remaining 6 superfeatures (dots, globules, lines, network structures, regression structures, and vessels), images in which at least 3 of 5 experts in study 1 had selected ≥1 of the subfeatures within the same superfeature as present were used as the gold standard for “superfeature present.” Images in which none of the 5 experts had identified any of the subfeatures within the same superfeature as present were used as the gold standard for “superfeature absent.”

### Nonexpert Crowd Annotations (Study 2)

To collect nonexpert image annotations, we used the commercially available platform DiagnosUs (Centaur Labs) [44] through a collaboration agreement. Users can sign up to the app and participate in competitions, which increases engagement and improves accuracy [37]. Users are recruited via a referral system or advertisements on social media. To ensure that only users somewhat skilled at a task computed average detection values, gold standard images were used for both training and validation. This left the remaining images, for which either 1 or 2 expert raters annotated a subfeature within the same superfeature as being present, as true test images. If a user did not reach at least 83% correctness for the validation items, that user’s choices were not used in the subsequent analysis. Each of the 6 superfeatures was presented as a separate task. In addition to the binary choice of presence or absence of a superfeature, we also collected reaction times to assess decision difficulty [45].

### Expert Crowd Annotations (Study 3)

As study 1 allowed experts to select from the 31 subfeatures, we replicated the methodology of study 2 to allow direct comparison with the nonexpert crowd. Experts in study 2 were dermatologists with ≥5 years of experience. We recruited 7 experts to use the DiagnosUs platform and annotate the same 248 images from studies 1 and 2 for the presence of the 6 superfeatures. For each of the features, we selected the first 5 dermatologists who completed annotation of the image set.

### Reaction Times

For each of the tasks in studies 2 and 3, we computed the per-item averaged logged reaction times as the log of (1 + reaction time) to approximate a normal distribution of measurement errors. These averaged logged reaction times were then regressed against the average responses and a quadratic term, allowing for an inverted-U–shaped response function, which peaked roughly at the (across-readers) point of indecision.

## Results

### Initial Expert Annotations (Study 1)

**Figure 1:**
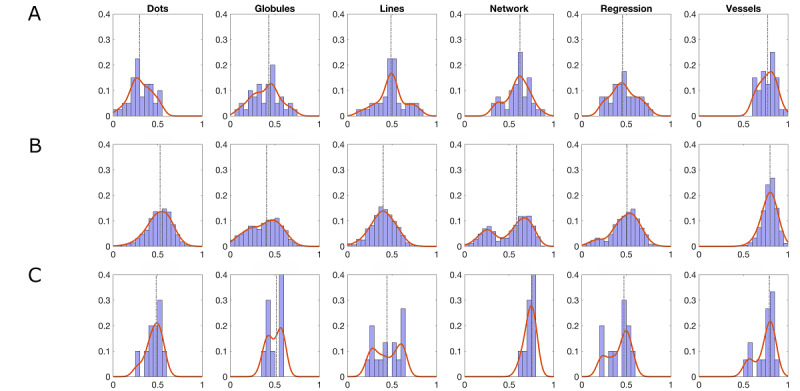
Pair-wise Cohen κ values for study 1 (A), study 2 (B), and study 3 (C).

In study 1, we found that dots showed poor agreement (median κ=0.298), whereas vessels showed excellent agreement (median κ=0.768). All other superfeatures showed fair to good agreement (Table 1). The resulting distributions of pairwise Cohen κ values are shown in Figure 1A. The number of resulting gold-standard images for each of the 6 superfeatures was as follows (0 readers/at least 3 readers, respectively): dots (93/61), globules (57/92), lines (129/60), network structures (63/140), regression structures (113/59), and vessels (152/66).

**Table 1:** Median Cohen κ values for pairwise readers. For study 2, pairs of readers were considered only if both readers saw at least 62 of the same images.

Feature	Study 1 (experts), median κ	Study 2 (nonexpert crowd), median κ	Study 3 (expert crowd), median κ
Dots	0.2977	0.5264	0.4829
Globules	0.4075	0.3945	0.5166
Lines	0.5205	0.3983	0.4433
Network structures	0.6175	0.5810	0.7575
Regression structures	0.4643	0.5066	0.4730
Vessels	0.7683	0.7977	0.7903

### Nonexpert Crowd Annotations (Study 2)

Providing demographic data pertaining to the users’ jobs and their reasons for using the DiagnosUs platform was optional; these data were collected from 190 users. Of these, 23 (12.1%) were physicians (2 dermatologists, 21 other specialties), 72 (37.9%) were medical students, 11 (5.8%) were nurse practitioners, 8 (4.2%) were physician assistants, and 76 (40%) were “other” or “other healthcare student.” The most common reason for using DiagnosUs was “improve my skills” (134/190, 70.5%), followed by “earn money” (37/190, 19.5%) and “compete with others” (19/190, 10%).

The number of users that engaged with each of the features varied for dots (92 users), globules (111 users), lines (82 users), network structures (97 users), regression structures (79 users), and vessels (95 users). Equally, the median number of ratings made per user per task varied for dots (160 images rated per user), globules (131 images), lines (177 images), network structures (91 images), regression structures (124 images), and vessels (104 images). The total number of crowd base ratings obtained in this study was 139,731, including 25,466 total ratings for dots, 40,853 for globules, 21,074 for lines, 17,114 for network structures, 17,020 for regression structures, and 18,204 for vessels.

The pattern we found in study 1 was largely replicated by the nonexperts. To ensure that there was sufficient and comparable overlap for images between pairs of readers, only pairs in which both readers saw at least 62 of the same images were evaluated. Dots and globules showed relatively lower agreement (with median κ values of 0.526 and 0.395, respectively), whereas network structures and vessels showed the highest agreement (with median κ values of 0.581 and 0.798, respectively). To allow a direct comparison between studies 1 and 2, we have compiled the 6 superfeatures into a panel figure (Figure 1A and 1B).

### Expert Crowd Annotations (Study 3)

Again, the patterns found in studies 1 and 2 were replicated, such that dots and globules showed relatively lower agreement (median κ values were 0.483 and 0.517, respectively), whereas network structures and vessels showed the highest agreement (median κ values were 0.758 and 0.790, respectively; Figure 1C).

We computed κ values for each nonexpert reader from study 2 compared to a single simulated expert by thresholding the responses in study 3 from 3 of 5 experts into a binary variable. The median κ values between nonexperts and the thresholded average expert reader were as follows: for dots, 0.709; for globules, 0.719; for lines, 0.714; for network structures, 0.838; for regression structures, 0.818; and for vessels, 0.728.

### Reaction Times

Irrespective of task, the reaction time varied by user (the median IQR for reaction time across users was 2.5 seconds to 4.3 seconds) and across images (the median IQR for the difference in reaction time per user was –0.93 seconds to +1.5 seconds), suggesting that the variability within users was somewhat greater than the variability across users.

**Figure 2:**
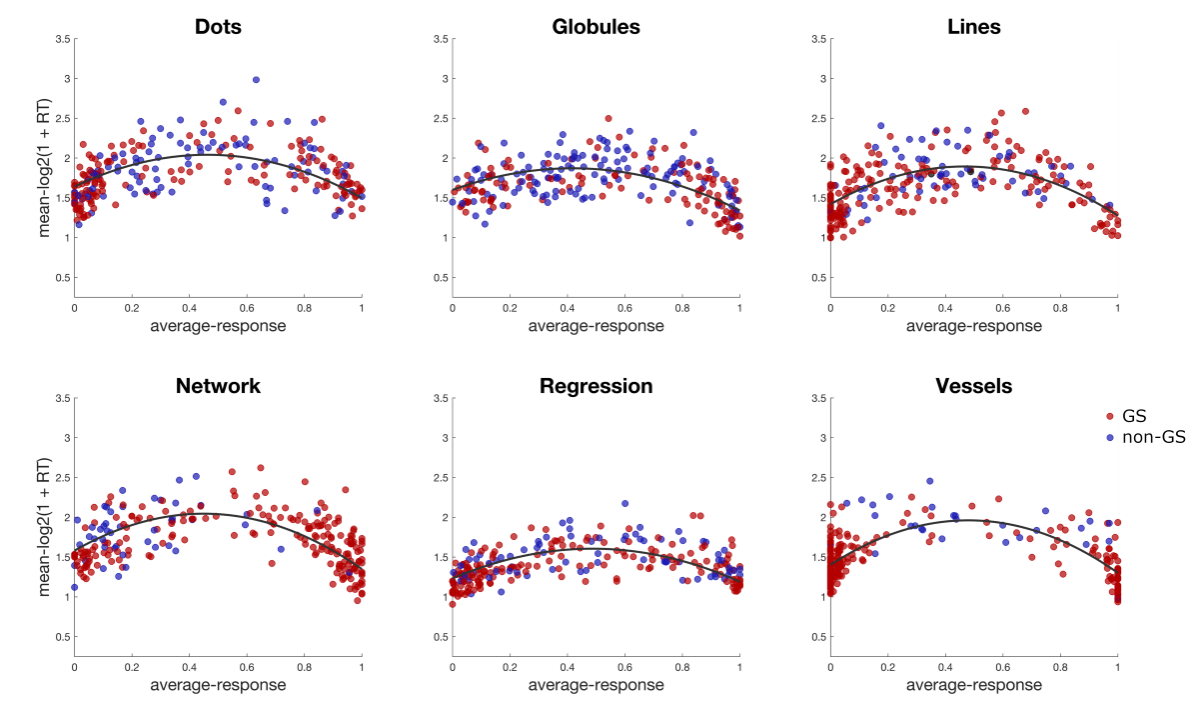
Log reaction times for gold standard images (shown by the red dots) and non–gold standard images (shown by the blue dots) of nonexperts regressed against their average responses and showing the estimated quadratic term for each superfeature. RT: reaction time; GS: gold standard.

For both the nonexperts and experts, the quadratic term accounting for the inverted-U–shaped response in averaged logged reaction times reached statistical significance across all tasks. Among the nonexperts, the *t* values (calculated with a 2-tailed *t* test) ranged from t_244_=–14.3 (for dots) to t_244_=–20.09 (for vessels). Among the experts, probably due to higher noise, the *t* values ranged from t_244_=–7.63 (for regression structures) to t_244_=–10.62 (for vessels). All *t* values were highly significant (*P*<.001). In all tasks and for both sets of readers, the linear term had a negative sign and was also significant (at lower levels), meaning that in all cases readers were faster to respond when a feature was present compared to when it was absent (Figure 2).

## Discussion

### Principal Findings

The main findings of this study confirmed the variable, and sometimes low, IRR between experts for identifying dermoscopic superfeatures on images of melanocytic lesions. The patterns of repeatability were mirrored in all 3 studies, highlighting that some features are more challenging to identify regardless of experience level. We found that the IRR between the untrained crowd and expert crowd was good to excellent for all superfeatures, suggesting that crowdsourced labels can be reliably used for future research. Reaction times were slower for lesions that would be considered more challenging in both cohorts, and therefore may be used as a proxy for decision difficulty.

### Initial Expert Annotations (Study 1)

In Study 1, the lowest level of agreement was observed for dots and globules, and the highest agreement was observed for network structures and vessels. This is in keeping with the findings of previous studies evaluating IRR for the identification of dermoscopic patterns among a group of experienced dermoscopists [10,46]. It has been suggested that poor agreement on criteria such as structureless areas, streaks, and dots or globules may be the result of lack of standardization in dermoscopy education [46,47].

Furthermore, the definition of dermoscopic structures may evolve over time. Whereas vascular structures and pigment network structures are easily recognizable, and their definitions have been consistent in the literature to date, dots and globules may be less easy to categorize. Tiny, numerous gray dots may be categorized as regression structures, and red dots may be defined as vascular structures [48-50]. Globules are defined as measuring >0.1 mm, which may be challenging to identify in dermoscopic images without a unit of measurement as a reference point. Going forward, it may be more feasible to consider dots and globules as a single criterion to eliminate the challenges encountered when attempting to differentiate them based on size.

### Nonexpert Crowd Annotations (Study 2)

A similar pattern of results was seen in study 2, suggesting that the gridlike pattern of a pigment network structure and the distinctive red color of vascular structures may be more repeatably identified by an untrained crowd. In keeping with the results of study 1, dots and globules were identified with poor repeatability. Again, this may be as a result of the ambiguity in distinguishing between the two on the basis of their diameter.

Prior studies have shown that dermoscopy by novice clinicians is no more accurate than visual inspection alone, and so an untrained crowd would not be expected to identify complex dermoscopic patterns, particularly when agreement between a group of world experts is known to be low, such as in our EASY study. To obtain reliable crowdsourced labels for complex medical images, an easier set of images may be used or participants may receive extended training; the study must also be designed to accommodate a large number of redundant labels [28]. In a study evaluating crowdsourcing as a method of identifying colonic polyps in CT colonoscopy images, McKenna et al [51] found that the crowd performance deteriorated with increasing difficulty, as well as with increasing reaction time. By collapsing the 31 subfeatures into 6 superfeatures, we created a more achievable task for a crowd with no prior experience of dermoscopy.

### Expert Crowd Annotations (Study 3)

The results from study 3 showed that agreement between experts was higher for dots, globules, and network structures when compared to study 1, in which annotations for subfeatures were aggregated into superfeature categories. It is known that there is a greater potential for disagreement with an increased number of categories and that the Cohen κ is typically observed to be lower in this circumstance [52]. Thus, if experts has been asked to choose from 6 superfeatures rather than 31 subfeatures, there would have been less potential for disagreement.

When comparing the median κ across all 3 studies, we found that repeatability for identifying all 6 superfeatures was similar across the experts and nonexperts. When comparing the median nonexpert annotations in study 2 to the thresholded expert annotations in study 3 for the same task, we saw that agreement was excellent for network structures and regression structures and good for the 4 remaining superfeatures. This suggests that the crowd was able to both repeatably and reliably identify dermoscopic superfeatures. Interestingly, agreement for vessels was higher within groups than between groups; thus, crowd annotations, although repeatable, were less accurate than expert annotations, suggesting that the crowd may be less reliable when annotating vessels. Vessels had the highest number of subfeatures (6) with distinct morphologies, several of which were not presented to the crowd during training on the DiagnosUs platform. Redesigning the tutorial may result in better accuracy for crowd annotations of vessels.

### Reaction Times

For both experts and nonexperts, there were 2 common patterns of response time (ie, the time it took a participant to feel confident enough to log a response varied as a function of estimated difficulty). For images for which the crowd showed low agreement (the average response was approximately 0.5 seconds), the response times were significantly slower than for images for which the crowd showed high agreement. For gold standard images (those for which ≥3 of 5 experts in study 1 agreed on the presence or absence of a feature) reaction times were faster than those for images of lesions upon which only 1 or 2 experts agreed, highlighting the challenging nature of these images. Furthermore, images where the feature was present had faster reaction times than those where the feature was absent, regardless of level of agreement. Overall, experts took longer to respond to images than nonexperts, suggesting that they exerted more effort to ensure a correct response. In addition, there was no financial reward for experts in this study; thus, they were less motivated to annotate as many lesions as possible within a designated timeframe.

### Limitations

One of the fundamental limitations of this study and future implications that can be drawn from it is the potentially low dependability of crowdsourced annotations. Although we found high repeatability and reliability of labels in study 3, this was for a relatively small set of images that had been carefully curated to have high-quality examples of a limited number of superfeatures.

There are a number of proposed methods to improve the quality of crowdsourced data. Crowd performance has been shown to improve with increased time spent training for the task, and participants that complete more readings have been observed to perform better [36,53]. Therefore, we may be able to improve performance of the crowd by providing additional training, as well as by increasing participant engagement, such as with greater financial rewards. This may, however, come at the cost of increased time and a smaller number of participants. Although crowdsourced annotations may be marginally less accurate than those provided by experts, the increased number of available labels for training ML algorithms has been shown to make them more robust to noisy data [54].

In this study, we validated the participants’ performance against gold standard images to ensure the quality of labels, and poorly performing participants were not included. In the absence of an expert-labeled image, DiagnosUs allows a ground truth to emerge with an unlabeled competition design in which images that show internal consistency across raters become the initial gold standard. Filtering of individuals may also be achieved by evaluating participants based on previously performed tasks or providing a pretask test [34]. Aggregating results via majority voting is another commonly used method of preprocessing to improve annotation quality. Annotations may also be evaluated by using them to train a ML model and using the model’s performance as a proxy for crowd performance [34].

It is essential that some level of quality assurance take place for crowdsourced annotations in the absence of expert labeling for comparison, as would be the case in future studies. Although agreement is traditionally considered an indicator of data reliability, it has been suggested that participants’ competence and confidence should be taken into account [55]. This can be achieved by filtering participants with poor accuracy on gold standard images, aggregating annotations, and using reaction time as a proxy for decision confidence. Images that give rise to long reaction times and a low level of agreement may then be transferred to an expert for annotation.

Many of the lesions in the archive are complex and have multiple dermoscopic patterns, which we observed created challenges for the experts to reliably identify, let alone the untrained crowd. Obtaining annotations for only 6 superfeatures may limit the diagnostic value of an ML tool. Crowdsourced labeling of the ISIC archive may be limited by its size; at the time this study was conducted, approximately 10,000 superfeature annotations were collected per day. However, engagement with the DiagnosUs platform continues to grow exponentially, and it currently receives in excess of 1 million crowd opinions daily across multiple tasks. Therefore, it may be entirely achievable to annotate the ISIC archive with crowdsourced labels within a timeframe of weeks to months.

Although the images in this study were subject to a manual quality assurance process, they were not standardized. For example, some images contained a unit of measure, which may have introduced bias when differentiating between dots and globules, as mentioned earlier in the discussion. 

Insufficient demographic data were collected by the DiagnosUs platform to allow meaningful subanalyses; however, disparities in experience level between users were highlighted. Importantly, 2 physicians specializing in dermatology participated in the crowd, and it therefore cannot be truly considered untrained. Due to the nature of the platform, it appeals to medical professionals as a learning tool with the aim of driving innovation in medical artificial intelligence, and the platform provides meaningful labels at scale regardless of the background of its users.

### Future Work

Given the sheer size of the ISIC archive, it would be infeasible to obtain annotations by expert dermoscopists for all images. We have shown the feasibility of obtaining crowdsourced annotations; this method can be used in several ways. First, it will allow hierarchical organization of the archive, allowing users to filter lesions based on dermoscopic patterns. Second, it may act as a teaching tool, allowing novice dermoscopists to learn patterns and corresponding diagnoses. And third, these annotated data may be used to develop novel ML tools. Even if only a small proportion of images are labeled by the crowd, a pattern classification or segmentation algorithm could be used to annotate additional images in the archive though a weakly supervised technique [56]. A hybrid crowd-algorithm approach has been successfully developed by several groups for the purpose of segmenting large databases of medical images [28,38,54,57].

The issues regarding “black box” algorithms have been raised as a barrier to implementation of these tools in clinical practice. Given the complexity of medical imaging data, a fully explainable algorithm would be unlikely to have adequate performance; however, use of interpretable outputs may go some way to assuage hesitancy in uptake. A classification tool that is also able to detect dermoscopic patterns that have influenced its decision would allow dermatologists to make more informed decisions when evaluating the output of the algorithm [22]. Furthermore, a multidimensional algorithm that is trained on both diagnoses and dermoscopic features may have increased accuracy when compared to those trained on diagnoses alone.

The next steps in exploring the applications of crowdsourced data are to expand labeling to a larger sample of images with a robust quality assurance process and incorporate the labels into a pattern-detection algorithm to be evaluated in a study of readers. Should this algorithm display acceptable performance measures, it may be deployed to label further images and be incorporated into a classification algorithm to improve its explainability.

## Appendix

Multimedia Appendix 1 Select dermoscopic features as presented to participants during the tutorial on the DiagnosUs smartphone app.

## Abbreviations

CT: computed tomography
EASY: Expert Agreement on the Presence and Spatial Location of Melanocytic Features in Dermoscopy
IRR: interrater reliability
ISIC: International Skin Imaging Collaboration
ML: machine learning
