# Supplementary material for: Agreement Between Experts and an Untrained Crowd for Identifying Dermoscopic Features Using a Gamified App: Reader Feasibility Study
Source: JMIR Med Inform. 2023 Jan 18;11:e38412. doi: 10.2196/38412 (PMC9892985; doi:10.2196/38412)
Supplement: Multimedia Appendix 1 [file medinform_v11i1e38412_app1.docx]

| **Feature** | **Descriptor and Image** | |
| --- | --- | --- |
| **Dots** | Small round structures measuring <0.1mm in diameter that may be black, brown, grey or bluish | |
| Dots: Regular  Regular dots are clustered at the center of a lesion or located on the network lines (left panel)  Dots: Irregular  Irregular dots are heterogenous in shape, size, color and are frequently found at the periphery of a given lesion or scattered throughout the lesion (right panel) | | 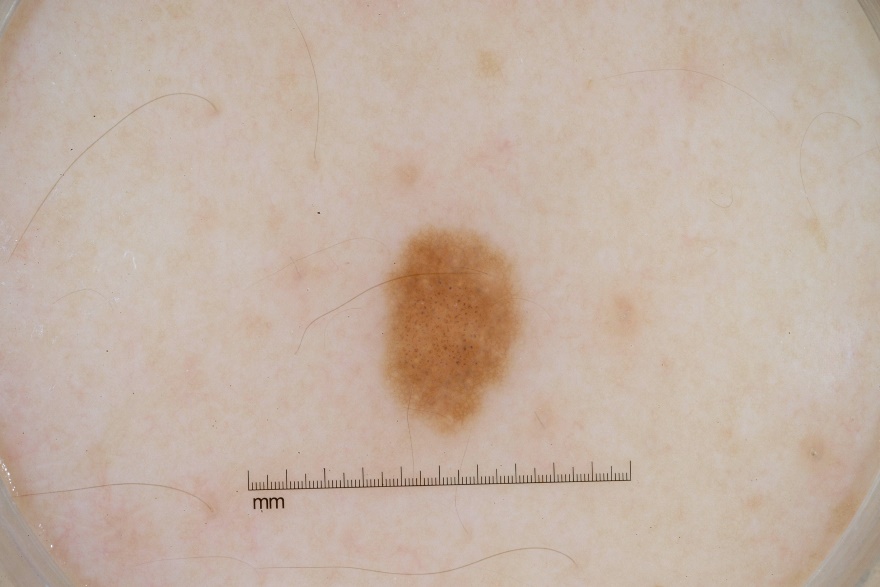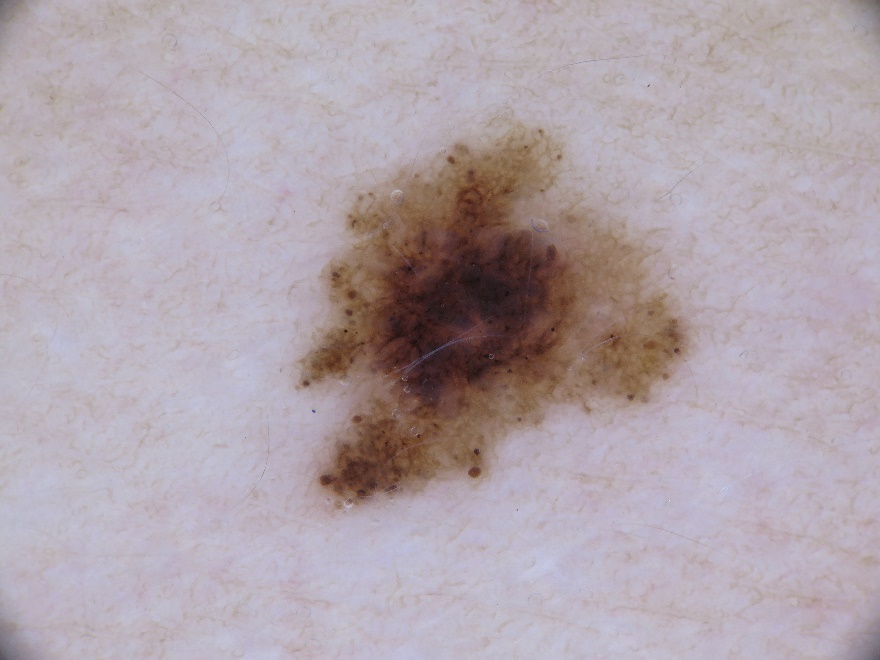 |
| **Globules** | Round to oval structures that may be brown, black, blue-gray, or red with diameters >0.1mm | |
| Globules: Regular  Regular globules are of similar size, shape and color, distributed symmetrically throughout the lesion (left panel)  Globules: Irregular  Irregular globules are of different size, shape and color, distributed in an asymmetric fashion (right panel) | | 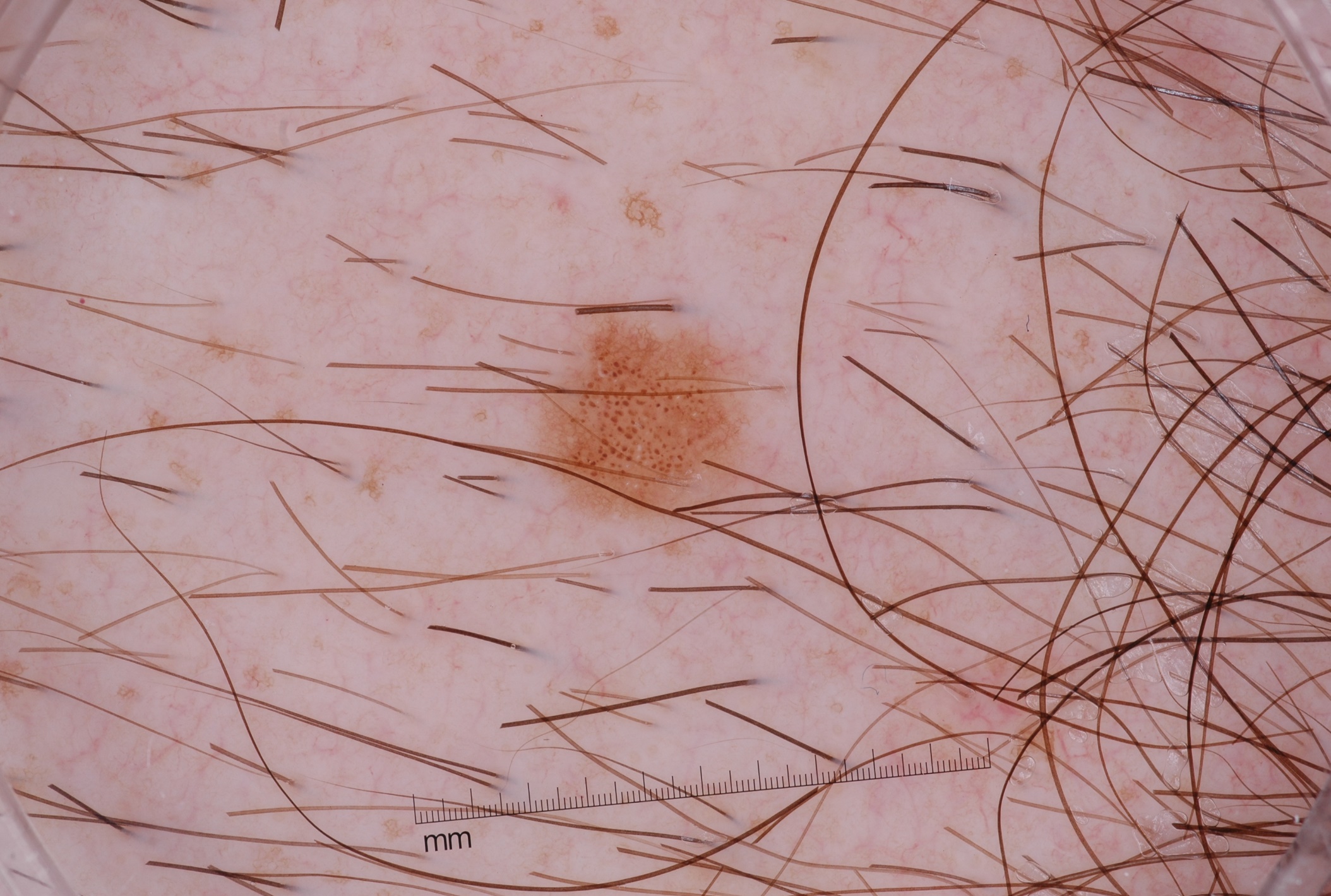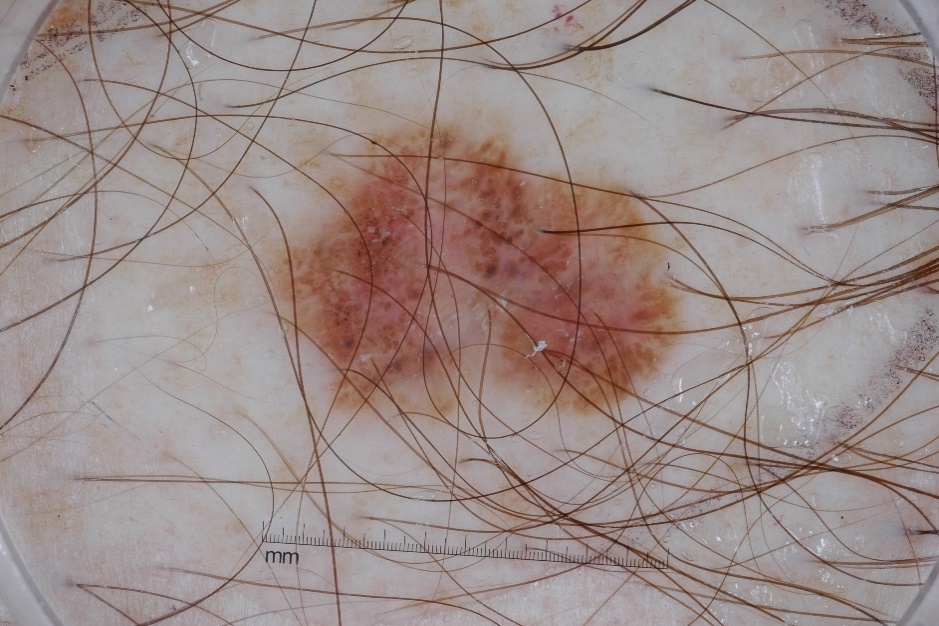 |
| **Lines** | | |
| Radial streaming | Linear pigmented projections seen at the periphery of lesions in a radial pattern | 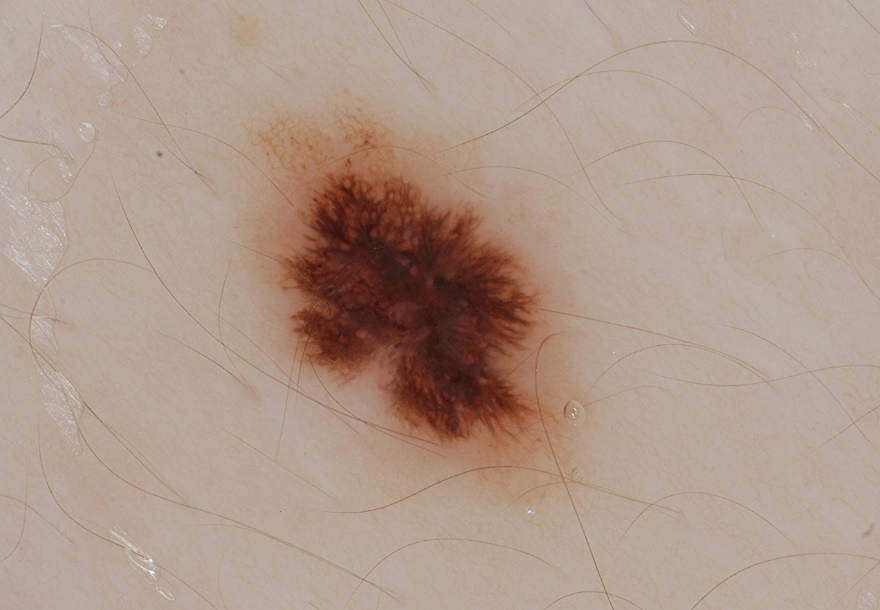 |
| Pseudopods | Streaks with bulbous projection at their tips. | 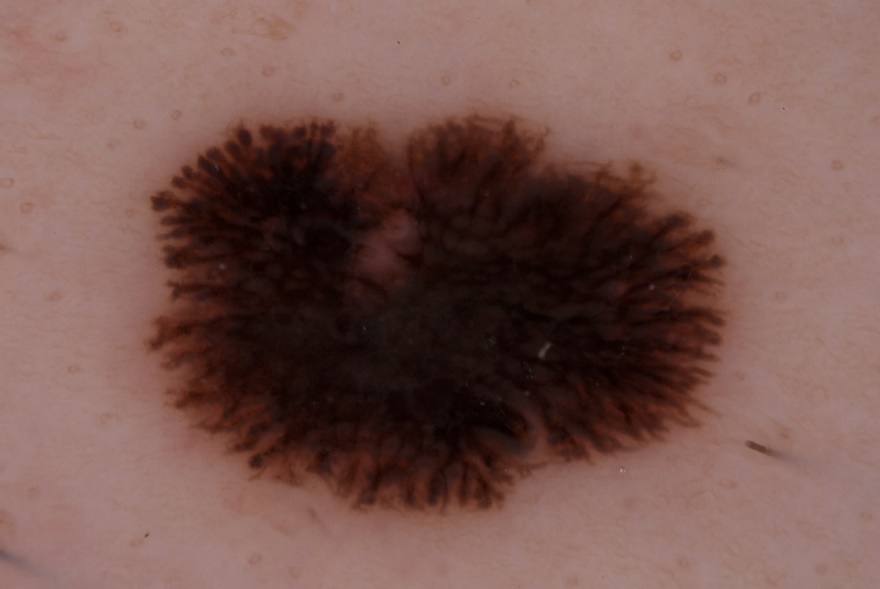 |
| Angulated lines | Geometric lines with a zigzag pattern, which may coalesce into polygons | 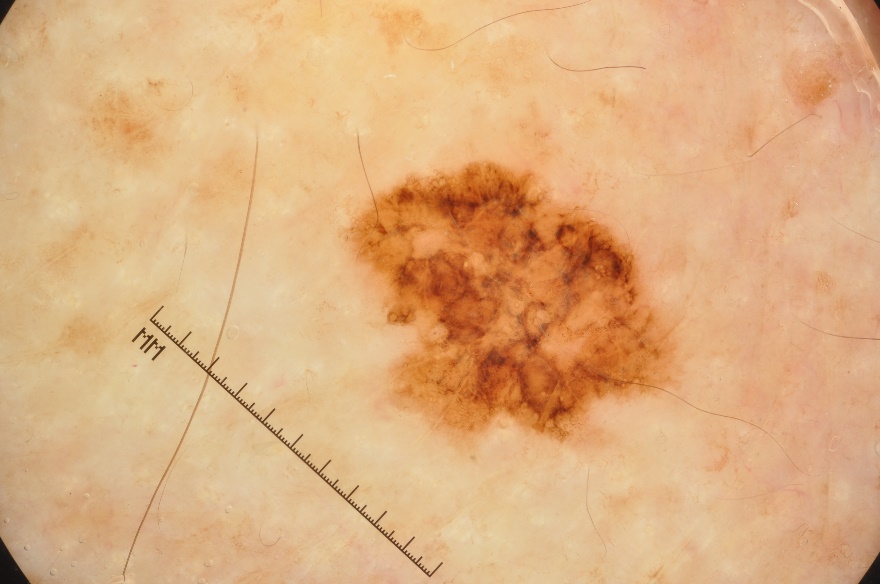 |
| **Network** | Pigment networks consist of a grid of intersecting pigmented “lines”, forming a honeycomb pattern. | |
| Typical network | Regularly meshed and composed of lines that are relatively uniform in width and homogenous in color. | 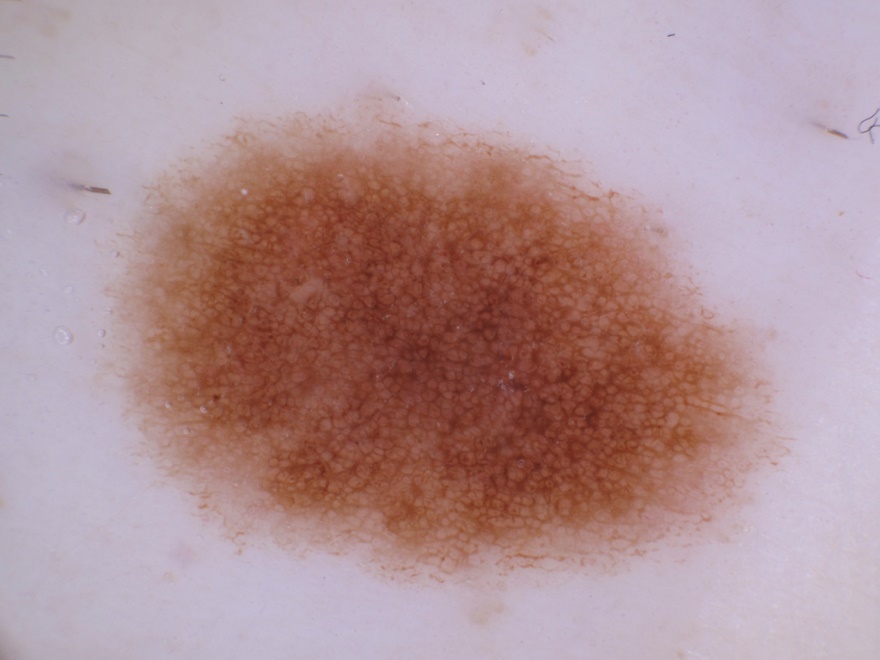 |
| Atypical network | Irregularly meshed with lines that vary in width and degree of pigmentation and with “holes” that are heterogenous in area and shape | 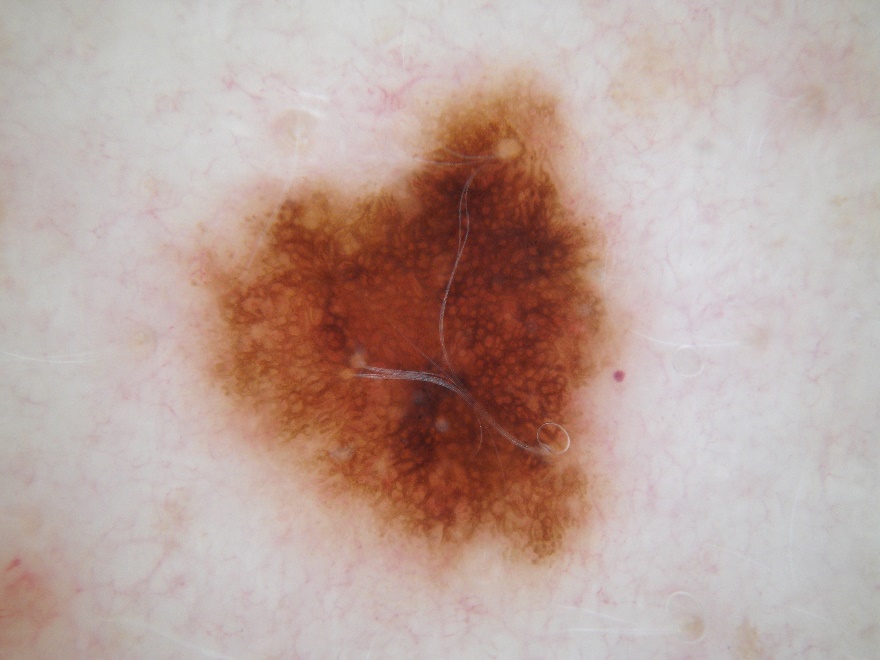 |
| Negative network | Relatively lighter areas comprising the apparent grid of the network and relatively darker areas filling the apparent “holes” | 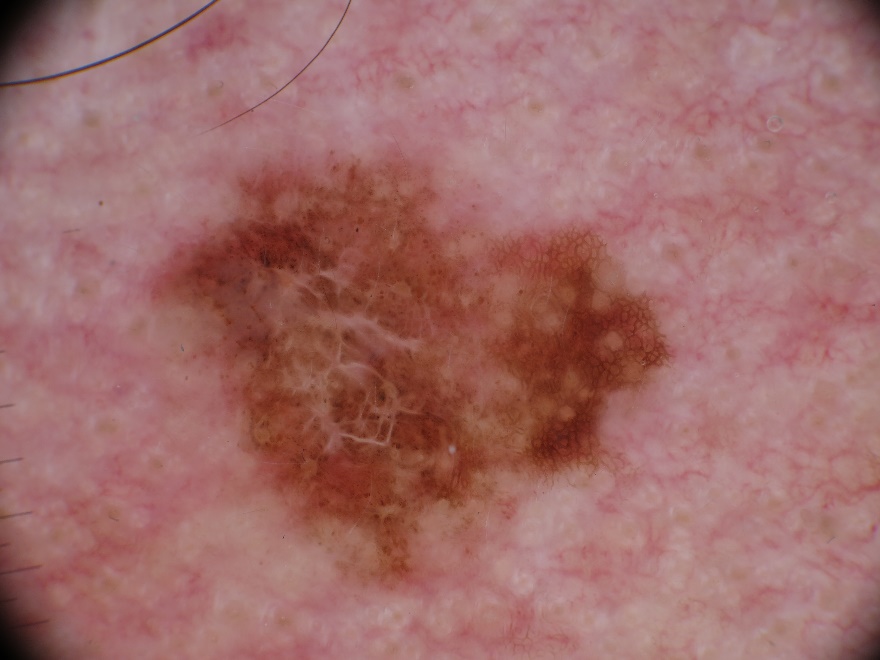 |
| **Regression** |  | |
| Scar-like depigmentation | White discoloration of the lesion (lighter than the surrounding normal skin) | 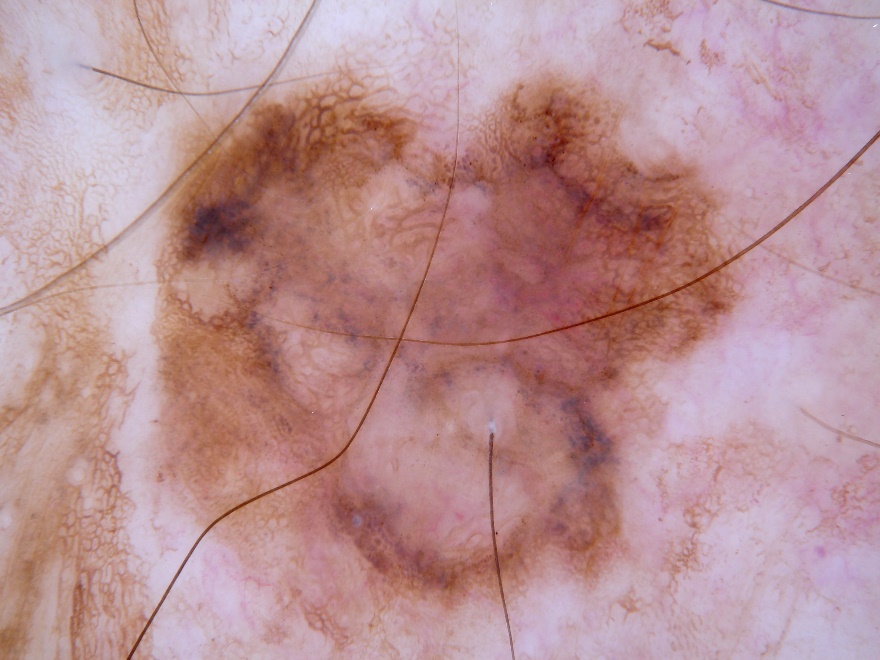 |
| Granularity/  peppering | Accumulation of multiple very small (<0.1mm), nondescript, blue-grey dots | 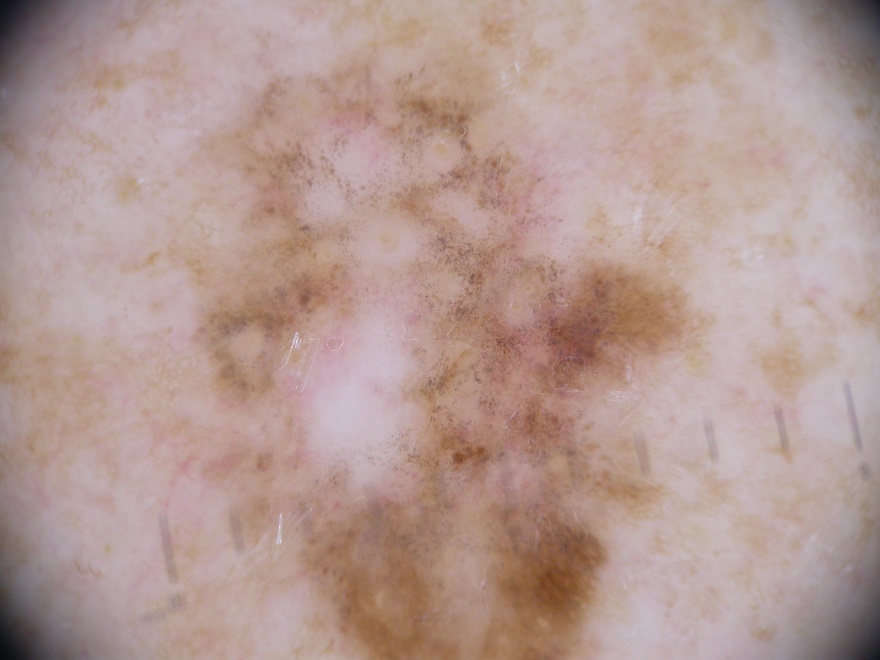 |
| **Vessels** | |  |
| Vessels | Bright red, branched vessels which are sharply in focus | 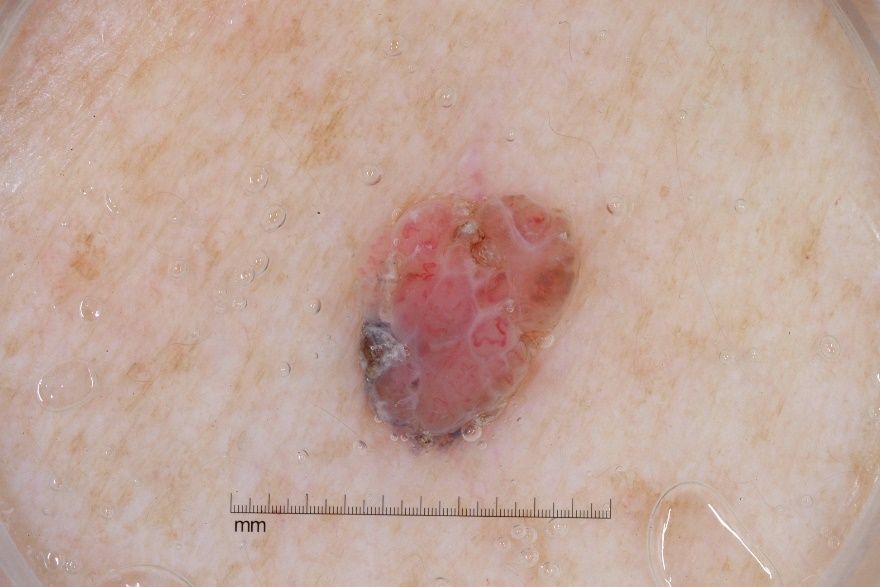 |
| Dotted vessels | Dotted vessels appear as small red dots with a diameter of 0.01-0.02mm | 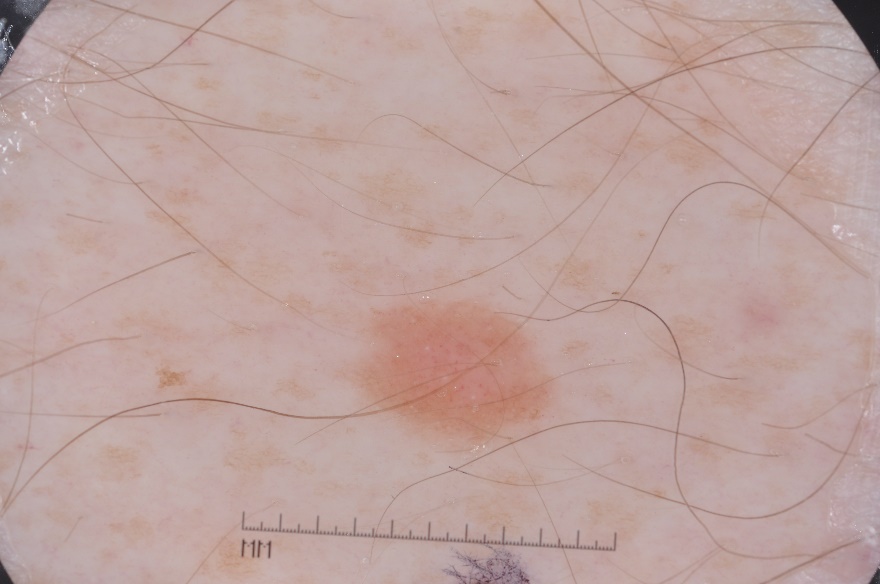 |
| Clods | Sharply demarcated roundish or oval areas with red, red-blue maroon, dark-red to black coloration. | 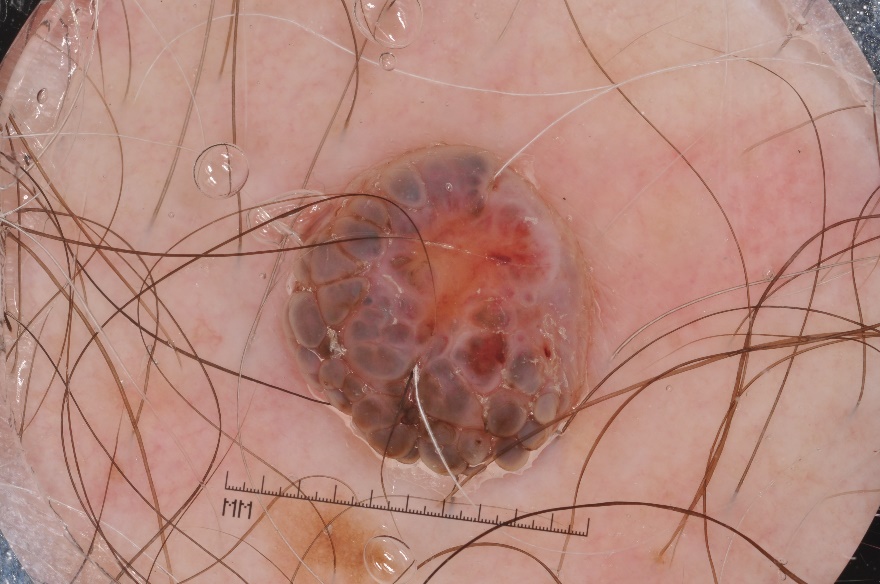 |
